# *Tinnea gombea* (Lamiaceae), a new species from the Sudanian savanna region, Nigeria based on integrative evidence

**DOI:** 10.1371/journal.pone.0280550

**Published:** 2023-03-16

**Authors:** Daniel A. Zhigila, Emmanuel I. Aigbokhan, A. Muthama Muasya

**Affiliations:** 1 Department of Biological Sciences, Bolus Herbarium, University of Cape Town, Cape Town, South Africa; 2 Department of Botany, Systematics Laboratory, Gombe State University, Gombe, Gombe State, Nigeria; 3 Department of Plant Biology and Biotechnology, University of Benin, Benin City, Edo State, Nigeria; National Cheng Kung University, TAIWAN

## Abstract

*Tinnea gombea*, endemic to the Sudan savanna grasslands in northern Nigeria, is described and illustrated. We used integrative evidence from morphological characters, ecology and molecular phylogenetic data. The new species is morphologically and ecologically similar to *T*. *barteri* and *T*. *aethiopica*, but can be readily delimited from these taxa by unique characters including a subshrub growth habit, leaves alternate to subopposite, blades lanceolate, apically acuminate, inflorescences raceme, bearing solitary flowers in upper leaf and bract axils, lilac to purplish dusky flowers and the inflated fruits dehiscent. The distribution and habitat of *T*. *gombea* are also distinctive, being restricted to the Sudan savanna, while the two most similar species are widespread in tropical Africa. Additionally, molecular phylogenetic assessments using nrITS and chloroplast *trnL-F*, *matK* and *rbcL* support the placement of *T*. *gombea* as a distinct species. *Tinnea gombea* is here assessed as Critically Endangered due to its small population size and restriction to a small area lacking conservation prioritization.

## Introduction

*Tinnea* Kotschy ex Hook.f, is a genus in the family Lamiaceae, subfamily Scutellarioideae, comprises 19 species native to the African continent [1, 2]. Species of the genus are found in the west, central and east tropical and southern subtropical regions occupying the savanna scrubs and the great Guinean forest. All species are narrow-ranged endemics except the widespread *T*. *aethiopica* Kotschy ex Hook.f. and *T*. *barteri* Gürke that extend from coastal areas of east Africa to tropical west and central Africa and to subtropical southern Africa [2]. The subtropical regions of southern Africa recorded the highest species diversity [3].

*Tinnea* species are erect annual or perennial herbs, suffrutices or shrubs arising from woody rootstock with quadrangular or terete stem transverse sections, covered with simple hairs; leaves typically simple, opposite or ternate, covered with unicellular to glandular trichomes on both adaxial and abaxial surfaces, with glands; bracts leaf-like but smaller; inflorescence racemose, in axils of upper leaves and bracts or forming terminal spikes; flowers typically pedicellate, solitary or cymose, upper part of the calyx 2-lipped, short and flat but 3-lipped, broad and spoon-shaped at lower side; becoming enlarged; and nutlets inflated, with conspicuous ridge of wings on the dorsal side [2].

The placement of the genus in Scutellarioideae was based on tuberculate corollas, exserted stamens and inflated fruits [1–3], and later was confirmed by phylogenetic studies of Li et al. [4] and Zhao et al. [5]. Robyns and Lebrun [1] separated the genus into two sections based on the floral arrangements namely, section *Sparsiflora* with solitary or 2–3-cymes flowers not forming distinct clusters and section *Spicata* with elongated spikes in axils of upper leaves and bracts or forming clusters at the terminal head. Section *Spicata* was further delimited into two subsections namely Scariosae (with two series) and Membranaceae based on calyx characters. The calyx in the former being scarious and the latter membranous. Although Robyns and Lebrun’s [1] monograph is the most comprehensive taxonomic treatments of *Tinnea*, the species delimitation and classification were completely artificial [5]. Therefore, the modern tools of integrative phylogenetics are required to test this hypothesis.

The rapid increase of genomic sequence data has made phylogenetics an indispensable tool for identification and classification of plants. The last two decades have witnessed growth in molecular phylogenetic studies in Lamiaceae [e.g. 6–8], at the subfamilial and tribal level [e.g. 9–12] as well as at generic levels [e.g., 13–15]. Although Li et al. [4] and Zhao et al. [5] included *Tinnea zambesiaca* Baker and *T*. *aethiopica* respectively in their molecular phylogenetic studies of Lamiaceae, assessments of species’ phylogenetic relationships within the genus *Tinnea* is lacking. According to Li et al. [4], the genus *Tinnea* is a close sister to *Scutellaria* L. The sampling of *Tinnea* species in these studies, however, was insufficient to allow assessing the monophyly of the genus and to contribute in understanding evolutionary relationships within *Tinnea* based on molecular phylogenetic analyses.

During field trips for the preparation of *a Field Guide to Herbaceous Plants of the Savanna* in Gombe State, Sudan savanna, northeastern Nigeria, we encountered noteworthy *Tinnea* populations on farmlands in Akko Local Government Area. However, we were unable to key the *Tinnea* collections to the descriptions from the *Flora of West Tropical Africa* [15], *Flora of Tropical Africa* [16], *a handbook of West African Weeds* [17], *Flora Zambesiaca* [18], *African Plant Database* [19] and Plants of the World Online [2]. After a detailed examination of the specimens, we hypothesized that these populations represent an undescribed species. Herein, we formally describe and illustrate this species new to science using morphological, ecological and molecular evidence.

## Materials and methods

### Morphological study

Morphological assessments were based on herbarium specimens and our field collections. Further, protologues of all published names in the genus *Tinnea* from Africa and all relevant taxonomic literature [1–3, 15, 16, 20] were consulted and reviewed. To delimit the potential new taxa from closely similar species, morphological variations were compared with herbarium (FHI, GSUH, K, S; acronyms follow [21]) specimens including type materials on JSTOR [22] and POWO [2]. A dissecting microscope (Leica GZ4) or stereomicroscope (Leica S9i) fitted with digital camera (Nikon DS-5M) and eyepiece micrometer were used to measure and photograph stems, leaves, inflorescences, flowers and fruits.

### Phylogenetic study

To assess the molecular phylogenetic placement of the new species within the genus *Tinnea*, three chloroplast DNA markers (*matK*, *rbcL*, *trnL-F*) and the nuclear ITS were used following [6, 12, 23]. Whole genomic DNA of the new species was extracted from 0.2 g of silica-gel dried leaves using the standard CTAB protocol [24] as amended by [23]. For detailed information on oligonucleotides and DNA markers, polymerase chain reaction mix, amplification thermal profiles and sequencing methods used, see our previous study [23]. Other sequences available for the genus *Tinnea* from other authors were downloaded from GenBank (https://www.ncbi.nlm.nih.gov/nuccor). Two species in the closest genus *Scutellaria* L. (Lamiaceae), *S*. *lateriflora* L. and *S*. *indica* Blume were selected as outgroups based on the Lamiaceae phylogenetic relationship hypothesized by [12] and [13]. Newly generated sequences were deposited on GenBank (see bold accession numbers, Table 1). Detailed information on accessions sampled for this study are provided in Table 1.

**Table 1:** Information on the accessions used in this study. GenBank numbers for new species are in bold. *matK*, *rbcL* OP454474 and OP454477, *trnL-F*: OP454489, the nuclear ITS.

	ITS	*trnL-F*	*matK*	*rbcL*	Collector	Herbarium	Country
*Scutellaria lateriflora* L.	MK356052	--	MK520635	MK526647	v0079576WIS	--	Korea
*S*. *indica* Blume	MH808599	MT265286	MN311856	MN192676	LuoY265	--	China
*Tinnea aethiopica* Kotschy ex Hook.f.,	--	--	KR735177	KR737558	E. DeFranco D2 K1214	EA	Kenya
*T*. *aethiopica*	--	--	KR735137	KR736734	E. DeFranco C1 K1214	EA	Kenya
*T*. *aethiopica*	--	--	KR734605	KR737516	E. DeFranco C2 K1214	EA	Kenya
*T*. *barbata* Vollesen	--	MT265260	JX518083	JX573048	G. Stafford 358	JRAU	South Africa
*T*. *barteri* Gürke	HQ911721		JX518083	HQ911385	Bendisks 050	--	--
*T*. *galpini* Briq.		MT265261	OV140630		Bester 8769	JRAU	South Africa
*T*. *gombea* Zhigila	--	--	**OP454468**	**OP454474**	D.A Zhigila 684	GSUH	Nigeria
*T*. *gombea* Zhigila	--	**OP454489**	--	**OP454477**	D.A Zhigila 685	GSUH	Nigeria
*T*. *gracilis* Gürke	--	HQ911722	HQ911386	--	A. Bjoernstad 1387	O	Tanzania
*T*. *rhodesiana* S.Moore	MF193549	MT265262	JX981418	JF265629	G. Stafford 359	JRAU	South Africa
*T*. *rhodesiana*	JF270971	MT265261	--	JX573049	G. Stafford 358	JRAU	South Africa
T. sp CP 278	MN257694	--	--	MN216517	C. Posthouwer 278	NHT	Tanzania
*T*. *zambesiaca* Baker	--	--	--	U28886	Bendisks 057	--	--

Five sequence datasets were generated: three chloroplast DNA datasets including, *matK*, *rbcL*, *trnL-F*, the nuclear ITS dataset and concatenated datasets (including all four DNA markers). Prior to the concatenation, the individual dataset was aligned using MAFFT Online service [25, 26] and manually checked and adjusted in BioEdit version 7.2.6 [27]. The individual and concatenated datasets were phylogenetically analyzed using the Bayesian Inference and Maximum Likelihood approaches using MrBayes version 3.2.6 x64 on XSEDE [28] and RAxML [29] as implemented in CIPRES cluster [1–3], respectively. For the nucleotide model substitutions of each DNA marker, the package jModelTest v.3.7 [30, 31] under the Akaike and information criteria (AIC) was independently selected. Thus, GTR+I+ Γ for ITS, GTR+ Γ for *matK* and GTR+I for *rbcL* and *trnL-F*. For details on the BI and ML analyses, refer to the phylogenetic work in [23] except that the Markov Chain Monte Carlo analyses for both BI and ML were run for 50,000,000 generations and sampling a tree at every 1,000th generation. The first 0.25 of sampled generations were discarded as “burn-ins” and the 50% majority-rule consensus tree was obtained from the remaining trees.

## Distribution map and conservation assessments

The distribution map was generated from specimen localities obtained from our field collections and herbarium voucher information using the naijR package version 0.4.0 as implemented in R version 4.2.0 [32]. Conservation status of a species is substantiated if one of the criteria A–E outlined in the guidelines of IUCN is established [33, 34]. Hence, the conservation status of the new species was assessed based on Criteria B (based on the area of occupancy and extent of occurrence), C and D (restricted, with a very small population size of <2,500 individuals and known only from the type locality) of the IUCN, [34]). The Geospatial Conservation Assessment Tool (GeoCAT; [35]; http://geocat.kew.org/) was used to quantify the Area of occupancy (AOO) and the extent of occurrence (EOO) with the default 2 × 2 km^2^ size following [36].

### Nomenclature

The electronic version of this article in Portable Document Format (PDF) in a work with an ISSN or ISBN will represent a published work according to the International Code of Nomenclature for algae, fungi, and plants, and hence the new names contained in the electronic publication of a PLOS ONE article are effectively published under that Code from the electronic edition alone, so there is no longer any need to provide printed copies.

In addition, new names contained in this work have been submitted to IPNI, from where they will be made available to the Global Names Index. The IPNI LSIDs can be resolved and the associated information viewed through any standard web browser by appending the LSID contained in this publication to the prefix http://ipni.org/. The online version of this work is archived and available from the following digital repositories PubMed Central, LOCKSS.

## Results

The statistic properties of the ITS, *trnL-F*, *matK*, *rbcL* and combined datasets are presented in Table 2. Even though the aligned sequence length for ITS was shorter than those of *trnL-F* and *matK*, the percentage of parsimony informative sites was significantly higher (32.1% versus 19.5% and 2.0% respectively). Further, the number of variable sites and consistency index recovered for each dataset reflect the lower phylogenetic signal in the chloroplast datasets versus the ITS. There are a number of shared alignment features between *Scutellaria* and *Tinnea*, e.g. a deletion (gap) in ITS, aligned position 103–132 that is not observed in any other *Tinnea* species. The already strong support values for a relationship between these two genera would have increased if gap coding had been used.

**Table 2:** Statistical properties of the dataset used for phylogenetic analyses.

Dataset	No. taxa	alignment length	PI	CI	variable sites
ITS	10	639	205 (32.1%)	0.564	0.74
*trnL-F*	8	785	153 (19.5%)	0.734	0.38
*matK*	12	756	15 (2.0%)	0.735	0.21
*rbcL*	13	548	10(1.8%)	0.538	0.10
Combined	15	2728	383(14.0%)	0.43	0.2144

PI = Number and percentage of parsimony informative site, CI = Consistency index minus uninformative sites.

**Fig 1:**
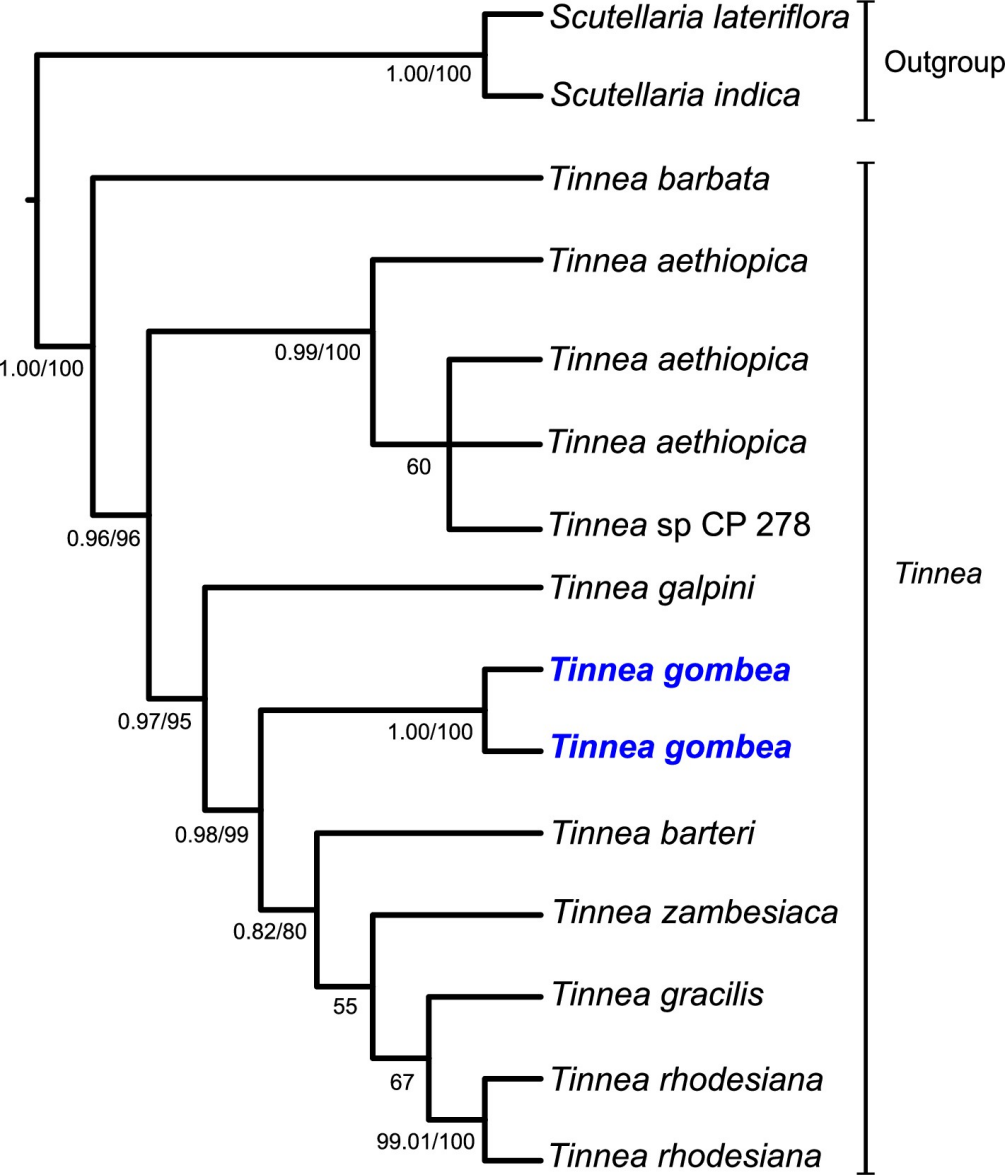
A 50% majority-rule consensus tree of the genus *Tinnea* obtained from the Bayesian analysis of the combined datasets of the nrITS, and *matK*, *rbcL* and *trnL-F*. Numbers on nodes indicate the posterior probability and the bootstrap support values of >0.80 and >55% respectively. Note that the new species (*T*. *gombea*) is in bold blue.

The molecular phylogenetic relationships among the Lamiaceae taxa as indicated in the ITS and chloroplast trees were highly congruent, although less resolved in the latter. Further, the tree topologies obtained from BI and ML analyses were congruent. Thus, only the 50% majority-rule consensus tree obtained from the Bayesian analysis is here presented (Fig 1). Using *Scutellaria* to root the tree, two strongly supported main clades are recovered: the first containing the outgroup *Scutellaria* species and the second containing the remaining *Tinnea* species. The monophyly of *Tinnea* was strongly supported as revealed by both bootstrap (BS) and posterior probability (PP) values of 100% and 1.00 respectively. The new species (*T*. *gombea*) is grouped with *T*. *barteri* with strong support values (BS = 99%; PP = 0.98) and *T*. *galpini* with moderate support value (BS = 95%; PP = 0.97).

**Fig 2:**
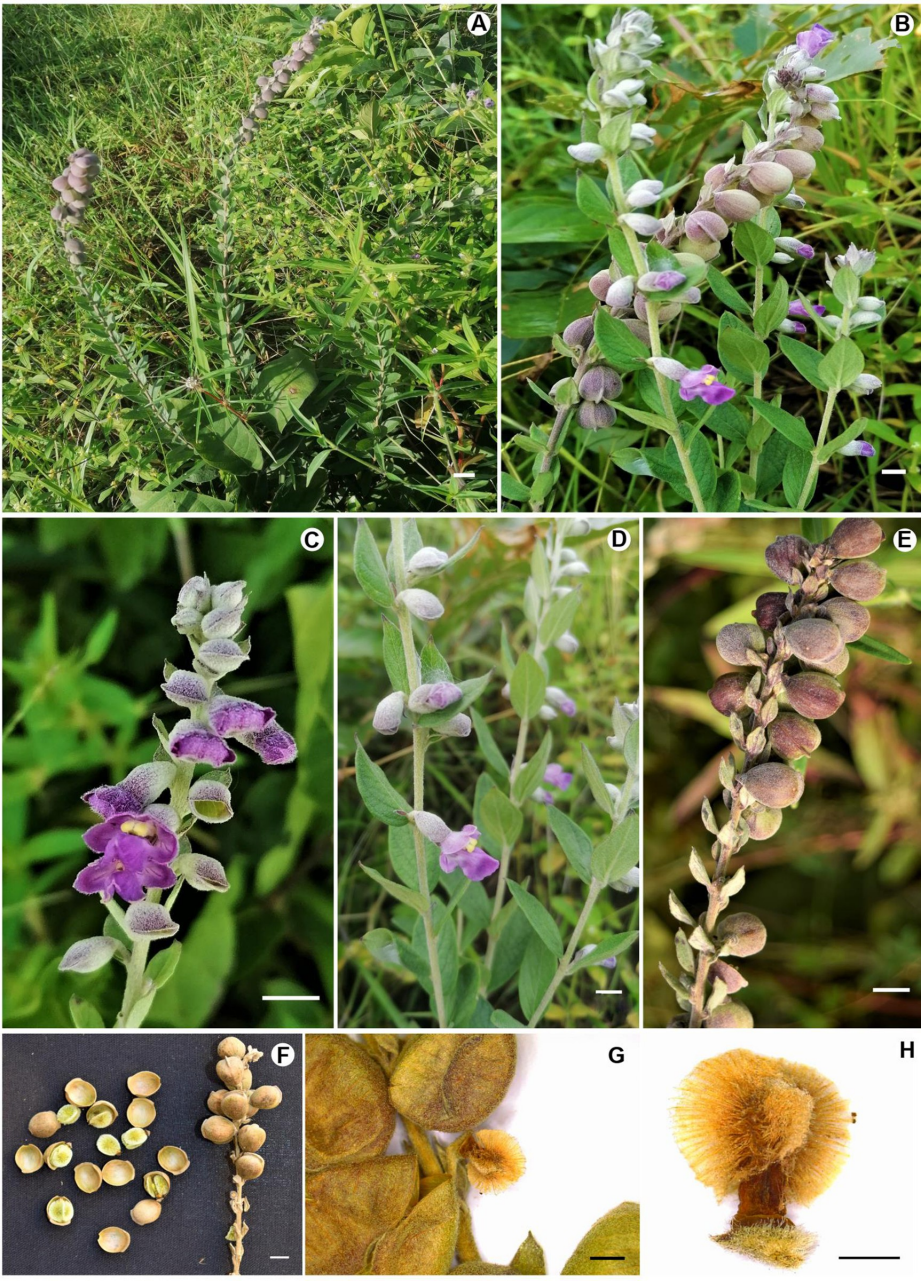
Morphological features of *Tinnea gombea*. (A) Plant habit and habitat; (B–D) young branches bearing vegetative and reproductive features; (E) Older branch with mature leaves, bracts and fruits; (G) closer view of dried fruits and leaves; (H) closer view of the fluffy seed with a tuft of basal hairs. Photos by D.A. Zhigila.

Morphologically (Table 3; Fig 2), *T*. *gombea* is characterized by erect growth habit, 20–50 cm tall, unbranched to lax branching pattern, from taproot system. The stems are single or 2–3 in number, terete, light green or glaucous appearance due to woolly hairs covering the stem and changes to tan with age. The leaves are alternate or subalternately arranged, attached to the stem by subsessile petioles, blade lanceolate, about 5 × 3 cm, covered with simple trichomes and conspicuous veins on both adaxial and abaxial surface, with abaxial surface usually light green, adaxial surface green basally obtuse, margins entire, apex acuminate. The racemose inflorescences have solitary flowers in upper leaf and bract axils, flowers with short pedicels, lilac to purplish grey, upper corolla lips shorter than the lower lips, with yellowish white stamens. The fruits are inflated, ovate to orbicular, about 10 × 8 mm, tan to greyish green, dorsally winged, and dehisces into two equal halves, 1-seeded.

**Table 3:** Morphological comparisons of *T*. *gombea* to congener species, *T*. *aethiopica*, *T*. *barteri* and *T*. *galpini*.

	*T*. *gombea*	*T*. *aethiopica*	*T*. *barteri*	*T*. *galpini*
Growth form	subshrub	shrub	herb	shrub
Plant height	<50 cm tall	>50 cm tall	>50 cm tall	>50 cm tall
Branching pattern	fastigiate	virgate	virgate	virgate
Branch density	lax-branched or single-stemmed	much-branched	lax-branched	lax-branched
Leaf attachment	alternate to subopposite	opposite to subopposite	opposite to subopposite	opposite to subopposite
Leaf blade	lanceolate	orbicular	orbicular, rarely obovate	lanceolate
Petiole length (mm)	0.2–0.5	5–10	2–5	3–12
Leaf apex	acuminate	apiculate	mucronate	apiculate or obtuse
Inflorescence type	raceme	spike	spike	spike
Pedicel length (mm)	2–5	5–15	5–10	5–15
Corolla colour	lilac to purplish grey	dark reddish brown or blackish purple	purple	dark purplish brown
Fruit	dehiscent	indehiscent	indehiscent	indehiscent
Distribution range	west tropical Africa	west, east and central tropical Africa	west and central tropical Africa	southern subtropical Africa

## Discussion

A detailed morphological comparison of the new species and other allied species of *Tinnea* was conducted (Table 3). *Tinnea gombea* is similar to *T*. *barteri* and *T*. *aethiopica* (Table 3; [15]). However, *Tinnea gombea* can be distinguished from both by a series of morphological traits such as having shorter petioles (usually to about 0.5 mm), leaves alternate to subalternately arranged, blade lanceolate, apically acuminate, shorter pedicels (usually <5 mm), lilac to purplish dusky flowers and fruits being dehiscent (versus both *T*. *aethiopica* and *T*. *barteri* having petioles ca. 5 mm long, orbicular or rarely long obovate leaves, apically apiculate and mucronate respectively, longer pedicels (usually >5 mm), purplish or reddish brown to blackish purple flowers and fruits being indehiscent). *Tinnea gombea* best fits into Robyns and Lebrun’s [1] section *Sparsiflora*. It shares the solitary flowers in the axils of the upper leaves with other species in this section. However, some characters in *T*. *gombea* overlap with species in section *Spicata* too. For example, elongated racemose inflorescences and flowers solitary or grouped as 2–5-cymes in bract axils are typical of species in section *Spicata* [1]. Although the floral arrangements were the main diagnostic character of sections in *Tinnea* [1], there were overlaps of these characters in species of the two sections.

Molecular phylogenetic analyses placed *T*. *gombea* as sister to a clade (Fig 1) comprising *T*. *barteri* and three southern African taxa (*T*. *zambesiaca*, *T*. *gracilis*, *T*. *rhodesiana*). The results of our phylogenetic analyses are generally congruent with the morphological and biogeographic patterns but do not support the infrageneric classification scheme proposed by [1].

**Fig 3:**
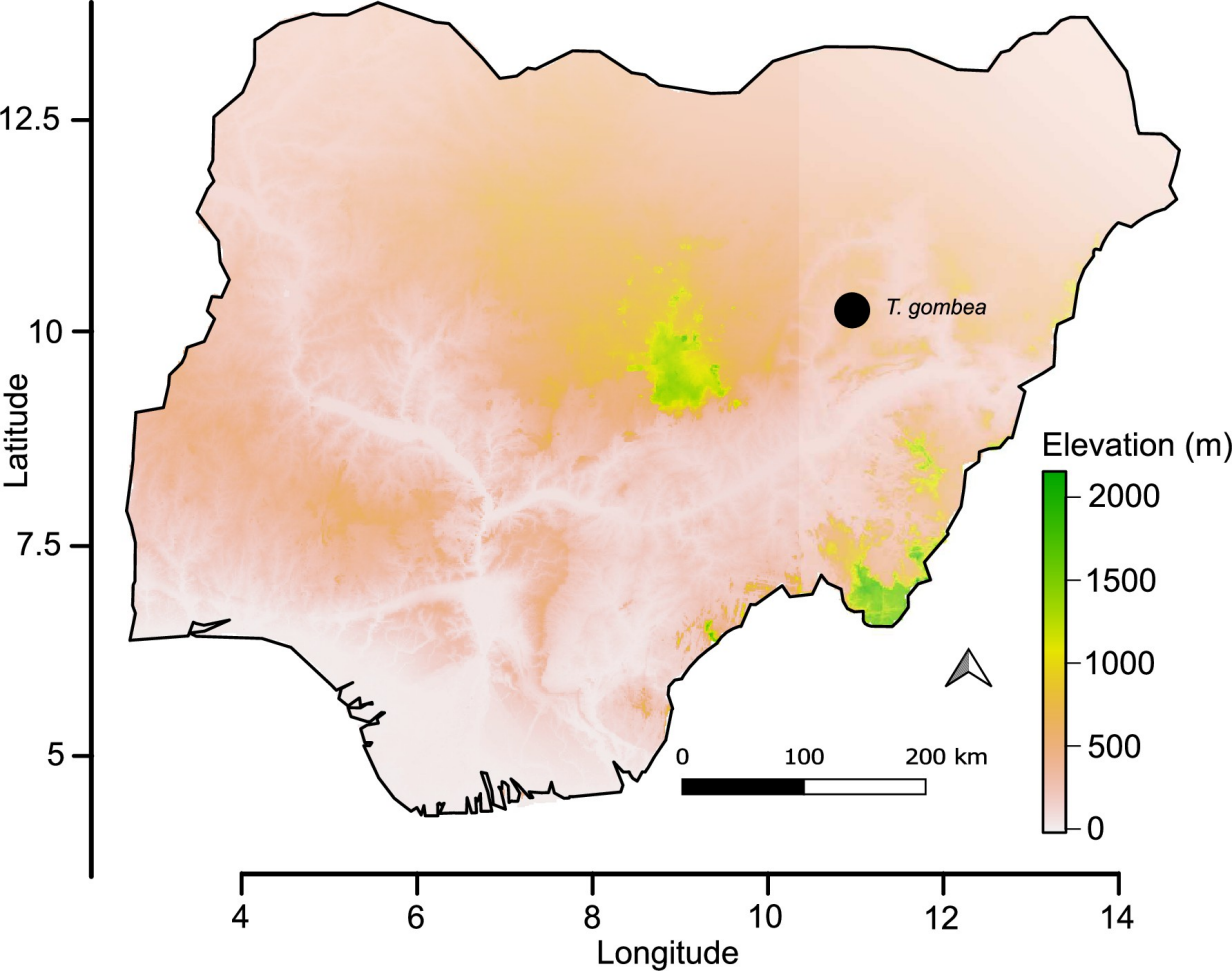
Distribution map of *Tinnea gombea* (black solid circle). The map was generated using naijR package version 0.4.0 as implemented in R version 4.2.0. and is therefore for illustrative purposes only.

In habitat, as for *T*. *barteri* and *T*. *aethiopica*, *T*. *gombea* also occurs in the savanna grasslands and woodlands of tropical Africa [1–3]. Within the savanna grasslands, however, *T*. *gombea* is restricted to the ecotones of loamy soil and rocky outcrops of the Sudanian savanna (Fig 3). It is noteworthy that these two species (*T*. *aethiopica* and *T*. *barteri*) were the only *Tinnea* reported previously in west tropical Africa [15].

**Fig 4:**
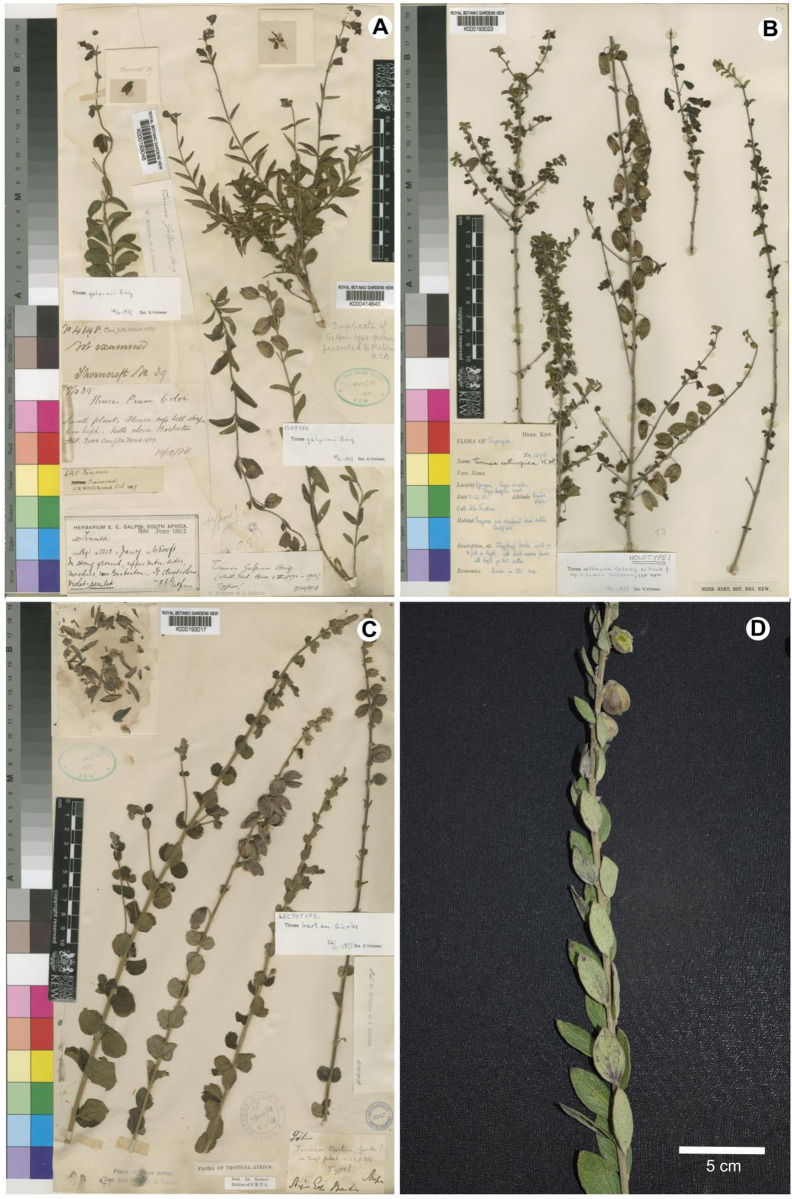
Scanned type herbarium specimens of the most similar *Tinnea* species. (A) *T*. *galpini*; (B) *T*. *aethiopica;* (C) *T*. *barteri*; and (D) *T*. *gombea* to show the similarities and variations in morphological characters. Copyright: the Board of Trustees of the Royal Botanic Gardens, Kew, United Kingdom. Reproduced with the consent of the Royal Botanic Gardens, Kew (K).

Thus, the integrative taxonomic evidence suggested by Sangster [37], here based on morphological variations (Figs 2 and 4; [1]), ecology and molecular phylogenetic position (Fig 1) strongly support the recognition of *T*. *gombea* as an evolutionarily independent and distinct taxon of a new species in *Tinnea*. Given the topology of our phylogenetic tree and the strong morphological and ecological evidences presented, a more robust taxon and DNA region samplings are recommended to reassess the infrageneric classification of *Tinnea*.

### Taxonomic treatment

#### *Tinnea gombea* Zhigila sp. nov. (Fig 2)

[urn:lsid:ipni.org:names:77311521–1] ***Type***

NIGERIA. Gombe State: Akko Local Government Area, in farmlands of the grassland Sudanian savanna, elevation 620 m, 10° 17’ 9.96"N, 11° 0’ 21.6"E, 19 September 2020, *D*.*A*. *Zhigila 685* (Holotype GSUH!, isotypes FHI!, K!).

#### Diagnosis

*Tinnea gombea* is most similar to *T*. *barteri* in branches being lax (2–5 in number), stems terete in cross-section, leaf blades minutely hairy on both sides, inflorescence racemose, calyx bladder-like and in ecological requirements; but differ in growth form being annual subshrub, to about 40 cm tall, branching pattern fastigiate (versus annual herb, >50 cm tall, virgate in *T*. *barteri*), leaf attachment alternate to 2-nate or subopposite, blade lanceolate, leaf apex acuminate in *T*. *gombea* (versus opposite, orbicular, mucronate in *T*. *barteri*), inflorescence racemose, solitary flowers in leaf and bract axils, anthers with basal hairs (versus spikes to 2–3-cymes, on long terminal head, anthers glabrous in *T*. *barteri*) and fruits dehiscent (versus indehiscent in *T*. *barteri*) (Table 3).

#### Description

Erect annual subshrub, 15–40 cm tall, main branches 1–4, lax, fastigiate branches, arising from a taproot system. ***Stems*** woolly, green to grey-green, sometimes purple tinged toward the upper portion, terete. ***Leaves*** alternate or subopposite to 2-nate, shortly (0.2–0.5 mm) petiolate to subsessile; blade narrowly ovate to lanceolate, 2–7 × 1.5–3 cm, straight or recurved, covered with dense indument on both adaxial and abaxial sides, veins conspicuous on the abaxial surface, inconspicuous at the adaxial surface, base obtuse, margins entire, apex acuminate. ***Inflorescences*** are long racemes; usually arranged unilaterally, flowers solitary and borne in bract axils, lax in the lower, clustered in the upper portion; ***bracts*** usually subtending flowers, leaf-like (but smaller, 1–2 ×1–1.5 cm wide), alternately arranged, blade ovate to lanceolate, recurved backward, woolly on both side, acuminate; peduncles ca. 0.5 mm long, tan. ***Flowers*** lilac to purplish grey; calyx two unequal pairs, 5–12 ×3–5 mm, upper lobes equal, short and rounded, lower lobes unequal, longer and rounded, densely woolly indument, membranous; corolla tube ca. 15 mm long, woolly; ***stamens*** 4, sessile to subsessile, in two unequal pairs, anthers yellowish, attached to the corolla tube by a tuft of woolly hairs; ***styles*** 10–12 mm long, recurved, with unequally bifid stigma. ***Fruits*** inflated and bladder-like, ovate to orbiculate, dorsally circular wings well-developed, ca. 10 ×8 mm including the wings, 10-veined, covered with woolly hairs, tan to greyish brown, dehisces into two valves on maturity, 1-seeded. ***Seeds*** ca. 5 mm in diameter, covered with long fluffy indument.

### Etymology

*Tinnea gombea* is named after its type locality, Gombe State, Nigeria.

### Phenology

*Tinnea gombea* flowers from August to September and fruiting between September and October.

### Distribution and habitat

*Tinnea gombea* is endemic to the Sudanian savanna and is currently known from Gombe State (Fig 3). Apparently, the species is uncommon within its area of occurrence in grasslands and woodlands of Sudanian savanna. Usually found on abandoned farmland together with various annual herbs and with perennial shrubs such as *Spermacoce* L. species, *Oldenlandia corymbosa* L., *Eragrostis tremula* Hochst., *Physalis angulata* L. and *Vernonia ambigua* Kotschy & Peyr. It usually grows at an elevation of about 620 m above sea level.

### Conservation assessments

*Tinnea gombea* is known from the type locality only. We recorded <100 mature individuals in each of the three subpopulations. We have made a concerted effort to sample the species from other potential localities within and around abandoned farmlands in the Sudan savanna but were unsuccessful. Therefore, further botanical surveys for possible localities for this species is recommended. At present, the area of occupancy (AOO) of 0.5 km^2^ and an extent of occurrence (EOO) of 5.00 km^2^ were estimated for the known subpopulations. We project a continuous decline in the AOO, EOO, number of subpopulations and number of mature individuals for *T*. *gombea* given the level of accelerated urbanization toward its area of occurrence. Further, flooding and agricultural activities (crop cultivation and overgrazing) are critical threats to this species. Hence, preliminarily, the red list status of Critically Endangered (CR; B1, B2 (b, c), C2 (a, b) and D) under the categories and criteria B–D of the IUCN [34] guideline is assigned to *T*. *gombea*.

### Additional specimen examined

NIGERIA. Gombe State: Akko Local Government Area, in farmlands of the grassland Sudanian savanna, elevation 620 m, 10° 17’ 6.11"N, 11° 1’ 55.6"E, 10 October 2020, *D*.*A*. *Zhigila 684* (GSUH!, FHI!, K!).
